# A Proteomics Signature of Mild Hypospadias: A Pilot Study

**DOI:** 10.3389/fped.2020.586287

**Published:** 2020-12-23

**Authors:** Coriness Piñeyro-Ruiz, Horacio Serrano, Inmaculada Jorge, Eric Miranda-Valentin, Marcos R. Pérez-Brayfield, Emilio Camafeita, Raquel Mesa, Jesús Vázquez, Juan Carlos Jorge

**Affiliations:** ^1^Department of Anatomy and Neurobiology, School of Medicine, University of Puerto Rico, San Juan, PR, United States; ^2^Department of Internal Medicine, School of Medicine, University of Puerto Rico, San Juan, PR, United States; ^3^Clinical Proteomics Laboratory, Internal Medicine Department, Comprehensive Cancer Center (CCC)-Medical Sciences Campus (MSC)-University of Puerto Rico (UPR), San Juan, PR, United States; ^4^Department of Biochemistry, School of Medicine, University of Puerto Rico, San Juan, PR, United States; ^5^Cardiovascular Proteomics Laboratory, Centro Nacional de Investigaciones Cardiovasculares (CNIC), Madrid, Spain; ^6^Centro de Investigación Biomédica en Red Enfermedades Cardiovasculares (CIBERCV), Madrid, Spain; ^7^Department of Surgery, Urology Section, School of Medicine, University of Puerto Rico, San Juan, PR, United States

**Keywords:** quantitative proteomics, etiology, proteomics, hypospadias, liquid chromatography-tandem mass spectrometry (LC-MS/MS)

## Abstract

**Background and Objective:** Mild hypospadias is a birth congenital condition characterized by the relocation of the male urethral meatus from its typical anatomical position near the tip of the glans penis, to a lower ventral position up to the brim of the glans corona, which can also be accompanied by foreskin ventral deficiency. For the most part, a limited number of cases have known etiology. We have followed a high-throughput proteomics approach to study the proteome in mild hypospadias patients.

**Methods:** Foreskin samples from patients with mild hypospadias were collected during urethroplasty, while control samples were collected during elective circumcision (*n* = 5/group). A high-throughput, quantitative proteomics approach based on multiplexed peptide stable isotope labeling (SIL) and liquid chromatography-tandem mass spectrometry (LC-MS/MS) analysis was used to ascertain protein abundance changes in hypospadias patients when compared to control samples.

**Results:** A total of 4,815 proteins were quantitated (2,522 with at least two unique peptides). One hundred and thirty-three proteins from patients with mild hypospadias showed significant abundance changes with respect to control samples, where 38 proteins were increased, and 95 proteins were decreased. Unbiased functional biological analysis revealed that both mitochondrial energy production and apoptotic signaling pathways were enriched in mild hypospadias.

**Conclusions:** This first comprehensive proteomics characterization of mild hypospadias shows molecular changes associated with essential cellular processes related to energy production and apoptosis. Further evaluation of the proteome may expand the search of novel candidates in the etiology of mild hypospadias and could also lead to the identification of biomarkers for this congenital urogenital condition.

## Introduction

Hypospadias is characterized by the localization of the urethral opening at any anatomical position on the ventral-medial side of the penis rather than near the tip of the glans. Its prevalence is ~21/10,000 live births (1–7). The most common type of this congenital urogenital condition is mild hypospadias (1st degree, anterior or Type I), comprising ~70% of cases, followed by moderate hypospadias (2nd degree, middle, or Type II) and severe hypospadias (3rd degree, posterior or Type III) with the remaining 30% (5, 8–11).

The formation of the male external genitalia is both a hormone-dependent and a hormone-independent multistep developmental event that spans embryonic and fetal periods. The androgen receptor (*AR*), activating transcription factor 3 (*ATF3*), estrogen receptor 1 and 2 (*ESR1 and ESR2*), steroid-5-alpha-reductase 2 (*SRD5A2*), hydroxysteroid (17-beta) dehydrogenase 3 (*HSD17B3*), hydroxy-delta-5-steroid dehydrogenase, 3 beta-and steroid delta isomerase 1 (*HSD3B1*), stAR-related lipid transfer domain containing 3 (*STARD3*), and mastermind like domain containing 1 (*MAMLD1*) are some of the genes that have been identified and associated with hypospadias (8, 12–25). In fact, molecular differences have been found at the gene, mRNA, and protein levels according to severity. Polymorphisms, mutations, and epigenetic changes in the *AR, CYR61, HSD17B3, HSD3B1, MAMLD1, SRD5A2*, and *STARD3* have been associated to hypospadias risk in severe types of hypospadias that have not been found in mild hypospadias (26–30). Some examples of these include reduced *AR* gene methylation (31) and increased GGN or CAG repeat sequence of the *AR* gene [(32, 33); respectively]. Allele variations and single nucleotide polymorphisms (SNPs) of the *SRD5A2* have also been associated with hypospadias risk (13, 27, 29, 34). Similarly, upregulation of *ATF3* in preputial tissue from hypospadiac boys has been reported (30, 35–37). SNPs and haplotypes in *ESR1* (17, 38, 39) and longer variants of (CA)n polymorphism in *ESR2* (40) have been identified and associated with hypospadias. At the mRNA and protein level, significant changes as severity increases have been observed in *AR, CYR61, ATF3*, and *CTGF*, some of which are estrogen-responsive genes (30, 41). In terms of downstream products, we found lower concentrations of aspartate in moderate and severe hypospadias but not in mild hypospadias (42). This finding is of interest as aspartate modulates steroidogenesis in Leydig cells (43–45). In addition, it has been shown that aspartate increases testosterone, dihydrotestosterone (DHT), luteinizing hormone (LH), and gonadotropin-releasing hormone (GnRH) levels, as well as *stAR* mRNA and protein expression levels and *AR* protein expression levels (46–50).

Despite these associations with hormone-dependent pathways, the etiology of hypospadias remains elusive. In addition to steroidal signaling, the development of the male external genitalia involves cell migration, differentiation, and proliferation, patterning, and apoptosis during development, all of which demand high energy inputs. For instance, luminization of the glandular urethra is achieved through apoptosis in the solid epithelial cord at the tip of the glans (51). Therefore, we hypothesized that disruption of proteins with key cellular roles previously ascribed to embryonic and fetal development would be apparent in the proteome of mild hypospadias. We found that cellular energy and apoptotic processes are disrupted in mild forms of this congenital condition.

## Materials and Methods

### Human Tissue Samples

This study was approved by the Institutional Review Board (IRB) under the Human Research Subjects Protection Office (HRSPO) at the University of Puerto Rico, Medical Sciences Campus. Parents of children undergoing urethroplasty and boys scheduled for elective circumcision were recruited at the HIMA San Pablo Hospital, Caguas, Puerto Rico. Hypospadias was assessed by Marcos Raymond Pérez-Brayfield, MD (pediatric urologist, Faculty in Surgery, Urology Section, School of Medicine, UPR). Informed consent was obtained from legal parents or guardians. Foreskin samples were collected from boys with non-comorbid mild hypospadias (also known as 1st degree, anterior or Type I hypospadias: *n* = 5) and elective circumcision (*n* = 5) between the ages of 5–28 months of age.

Mild hypospadias cases were confirmed during urethroplasty and included two glandular, one coronal, and two subcoronal cases after penile degloving and chordee release whenever present. Table 1 shows the age of the patient and the location of the urethral meatus. Given that this study did not control for the age of the patient at the time of the urethroplasty procedure for mild hypospadias cases, the age distribution in this patient cohort was not normally distributed. Mann-Whitney test revealed a significant age difference between experimental groups (*p* = 0.02; see Discussion for further details). Samples were obtained from both lateral and dorsal inner foreskin tissue, which is mostly comprised of mucous membrane. Samples with suggestive clinical changes of balanitis xerotica obliterans (*BXO*) were excluded from the study.

**Table 1:** Clinical characteristics of patients at the time of urethroplasty.

	**Patient**	**Age (months)**	**Urethral meatus location**
Controls	C1	8	Typical
	C2	24	Typical
	C3	28	Typical
	C4	28	Typical
	C5	11	Typical
Mild hypospadias	H1	22	Glandular
	H2	6	Subcoronal
	H3	5	Glandular
	H4	6	Coronal
	H5	6	Subcoronal

### Preparation of Protein Extracts

One hundred milligrams of wet foreskin tissue were weighed and homogenized with a tissue tearor in 1.0 mL of Lysis Buffer (50 mM Tris, 10 mM DTT, and 1% SDS pH 6.8). Samples were incubated for 5 min at 97°C with shaking (600 rpm, Eppendorf Thermomixer), and thereafter incubated overnight in rotator at 4°C. Homogenates were centrifuged for 5 min at 13,000 rpm, and the supernatant was collected. Protein concentration in the protein extracts was measured using the RC DC Protein Assay Kit II (Bio-Rad, USA) following the manufacturer's instructions.

#### Protein Digestion and Peptide Labeling

Protein extracts (100 μg) were concentrated on 30 K Filter-Aided Sample Preparation (FASP) filters (Expedeon). After washing with denaturing buffer (8 M urea in 100 mM Tris-HCl pH 8.5) at 10,000 rpm for 15 min, free thiol groups were alkylated by incubation with 50 mM iodoacetamide 30 min at room temperature in the dark. Then the filters were washed with denaturing buffer followed by washing with trypsin digestion buffer (50 mM ammonium bicarbonate pH 8.8). Protein samples were digested overnight at 37°C with sequencing grade trypsin (Promega, Madison, WI, USA) at 1:40 (w/w) trypsin: protein ratio in digestion buffer. The resulting tryptic peptides from each sample were recovered by centrifugation at 10,000 rpm for 5 min after the addition of 40 μl of trypsin digestion buffer, after which 50 μl of 500 mM NaCl was added and the filters centrifuged for 15 min at 10,000 rpm. Trifluoroacetic acid was added to a final concentration of 1%, and the peptides were desalted on C18 Oasis HLB extraction cartridges (Waters Corporation, Milford, MA, USA) and dried-down. Peptide labeling was performed using a 10-plex TMT kit (Tandem Mass Tag, Thermo Scientific, USA) according to the manufacturer's instructions, and desalted onto Oasis C18 cartridges before analysis by liquid chromatography-tandem mass spectrometry (LC-MS/MS).

#### Protein Identification by Liquid Chromatography-Tandem Mass Spectrometry Analysis

LC-MS/MS was performed using a nano-HPLC Easy nLC 1,000 chromatograph coupled to a Q-Exactive hybrid quadrupole orbitrap mass spectrometer (Thermo Scientific). Peptides were loaded onto a C18 RP nano-precolumn (75 μ m I.D. and 2 cm, Acclaim PepMap 100, Thermo Scientific), and separated on an analytical C18 nano-column (75 μ m I.D. and 50 cm, Acclaim PepMap 100) using a continuous gradient consisting of 8–30% B for 60 min and 30–90% B for 2 min (*B* = 90% ACN, 0.1% formic acid) at 200 nL/min. The chromatographic run acquired an FT-resolution spectrum (140,000 resolution) in the 390-1,500 m/z range, followed by data-dependent MS/MS spectra of the 15 most intense parent ions. The normalized HCD collision energy was set to 30%, and the parent ion mass isolation width was 1.5 Da.

Peptides were identified from MS/MS spectra using the SEQUEST HT algorithm integrated with Proteome Discoverer 2.1 (Thermo Scientific). MS/MS scans were matched against a human protein database (UniProt 2017_12_19 Release). Parameters were selected as follows: two trypsin missed cleavages maximum, 800 ppm precursor mass tolerance and 0.02 Da fragment mass tolerance. Cys carbamidomethylation, N-terminal TMT labeling, and Lys TMT labeling were chosen as fixed modifications, while Met oxidation was considered a dynamic modification. For peptide false discovery rate (FDR) assessment using the refined method (52, 53), MS/MS spectra were searched against the corresponding inverted human database.

#### Quantification of Protein Abundance Changes

**Figure 1:**
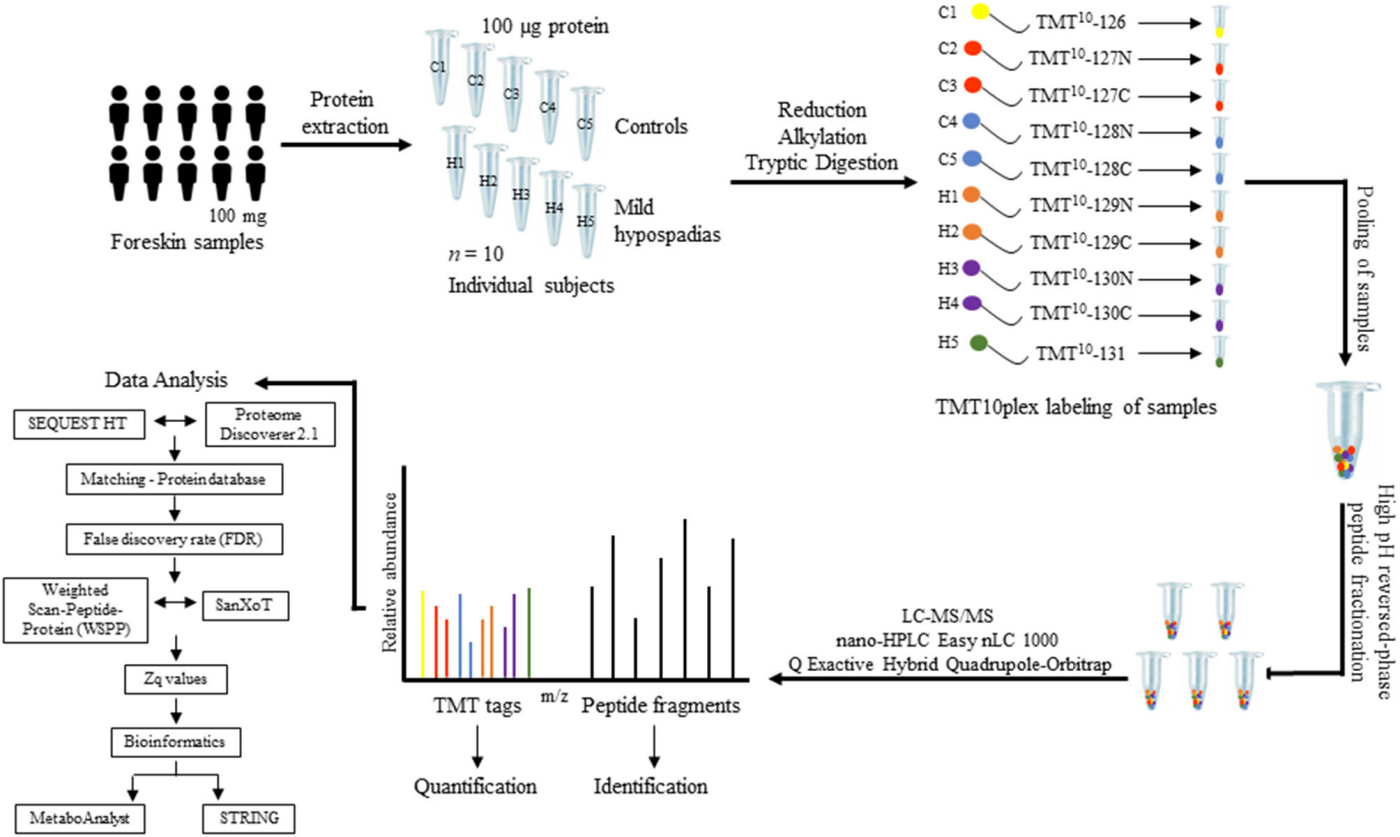
Proteomics study design. Foreskin samples were collected during scheduled urethroplasty from patients with mild hypospadias (*n* = 5) or from individuals submitted to a circumcision procedure (control group, *n* = 5). One-hundred milligrams of foreskin samples from each patient were homogenized and proteins extracted from tissue. A total of 100 μg of protein was processed per sample. Protein extracts were digested with trypsin using the FASP technology. Subsequently, peptide samples were labeled using a 10-plex TMT kit. Each sample was labeled with a specific TMT mass tag. After labeling, samples were pooled and separated into five fractions using high pH reversed-phase peptide fractionation. Peptides were resuspended in 0.1% formic acid and analyzed by LC-MS/MS. Upon fragmentation, the specific reporter ion released from each of the 10 mass tags shows in the low-mass region of the MS/MS spectrum. The intensity of this reporter ion was used to measure relative peptide abundance levels among samples, from which relative protein abundance between control and mild hypospadias cases was inferred (see text for details).

Quantitative information was extracted from the intensity of the TMT reporter ions in MS/MS spectra. Protein abundance changes were assessed based on the Weighted Scan-Peptide-Protein (WSPP) statistical model (54) under the SanXoT software package (55). This model provides the standardized variable Zq, which is defined as the mean-corrected log2-ratio expressed in units of standard deviation at the protein level. Thus, Zq values were calculated for each sample, both mild hypospadias, and control samples, with respect to a weighted average of control samples. The study design is depicted in Figure 1.

#### Statistical Analyses

Statistical comparison between hypospadias and control group Zq values was performed by Student's *t*-test. A *p*-value < 0.05 was considered significant. The MetaboAnalyst (56) online platform was used with Zq values to perform multivariate statistical analyses, including principal component analysis (PCA) and partial least squares-discriminant analysis (PLS-DA).

#### Functional Enrichment Analysis

The STRING database (http://string-db.org), version 11.0 (57), was used for functional enrichment analysis with proteins found significantly decreased (95 proteins) or increased (38 proteins) in the hypospadias group. A low protein-protein interaction (PPI) enrichment *p*-value indicates that the nodes are not random and that the observed number of edges is significant. A 5% FDR cutoff was used to evaluate significant functional category enrichment. Supplementary Table 1 details accession numbers, gene, and protein names for these proteins.

#### Validation of Cytochrome C by ELISA

Cytochrome C was detected and quantified by enzyme-linked immunosorbent assay (ELISA; *n* = 10 controls and *n* = 8 mild hypospadias). Test results were submitted to the Mann-Whitney U-test statistical analysis. Human Cytochrome C Platinum ELISA (Thermo Fisher Scientific, Waltham, MA) was used for quantitative detection following the protocol provided by the manufacturer.

## Results

### Proteomic Profile of Mild Hypospadias

**Figure 2:**
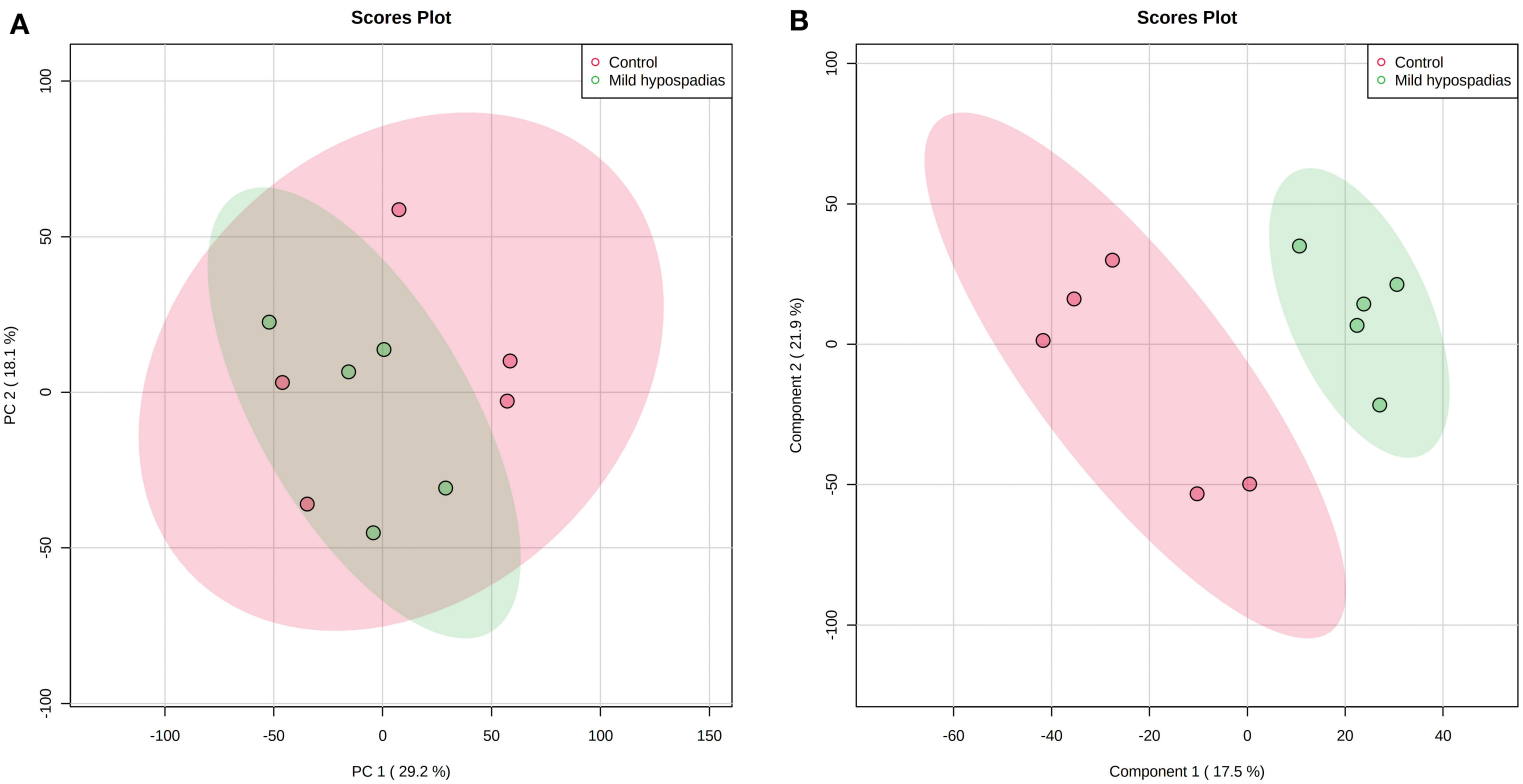
Mild hypospadias protein profile differs from controls. Zq values from a total of 2,522 proteins identified with two or more peptides were subjected to PCA and PLS-DA. **(A)** Score plot for PC1 (29.2% variance explained) vs. PC2 (18.1% variance explained). **(B)** Score plot for PLS1 (17.5% variance explained) vs. PLS2 (21.9% variance explained). The ellipses illustrate 95% confidence region for these groups. *n* = 5/group.

**Figure 3:**
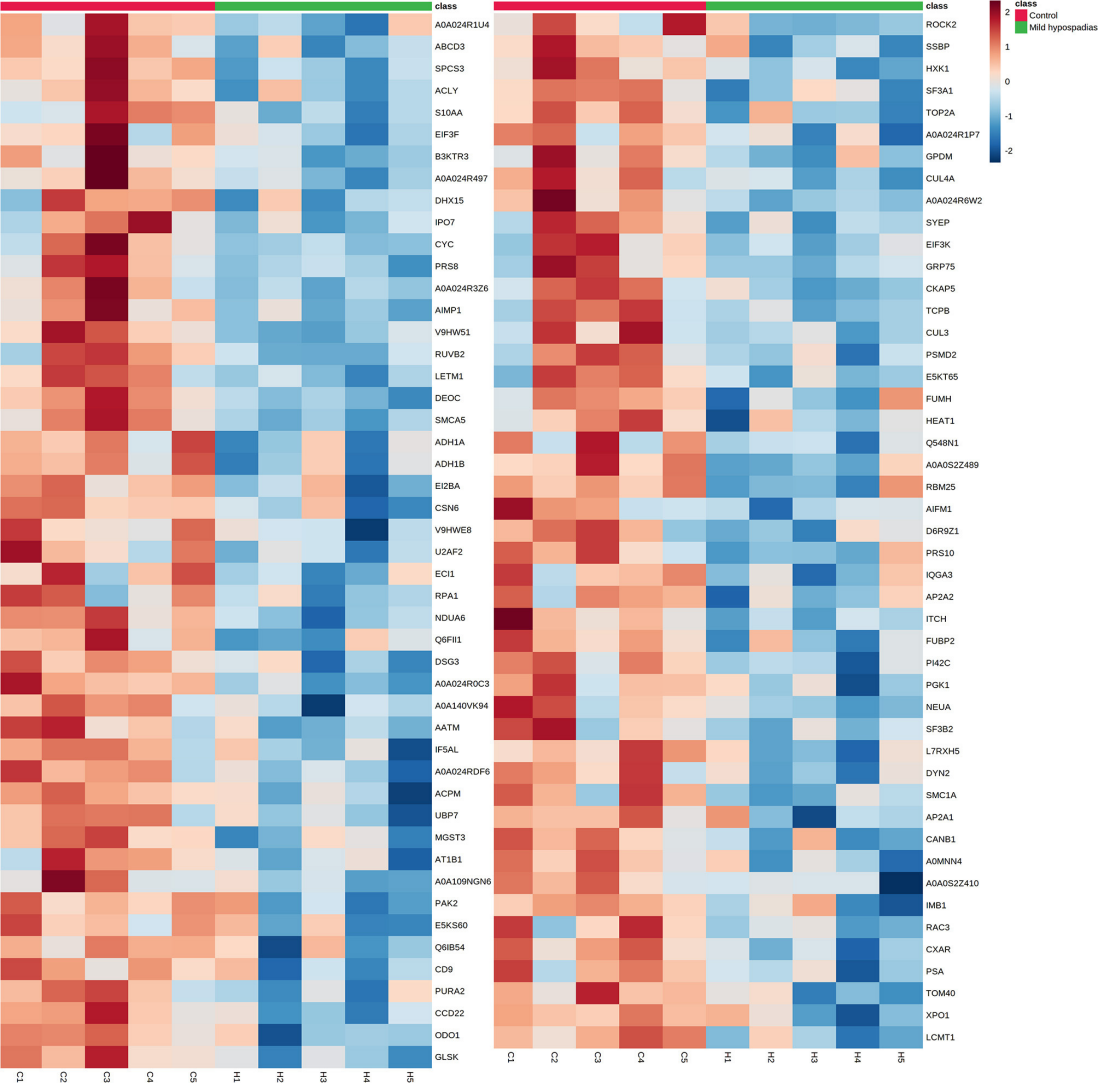
Proteins found downregulated in the proteome from mild hypospadias patients. Ninety-five proteins were found decreased between control and mild hypospadias samples. Heatmaps depict protein abundance changes in terms of Zq across samples (C1-C5 for control and H1-H5 for mild hypospadias samples). The magnitude of abundance change [increased (red) or decreased (blue)] is shown according to the color scale on the right.

**Figure 4:**
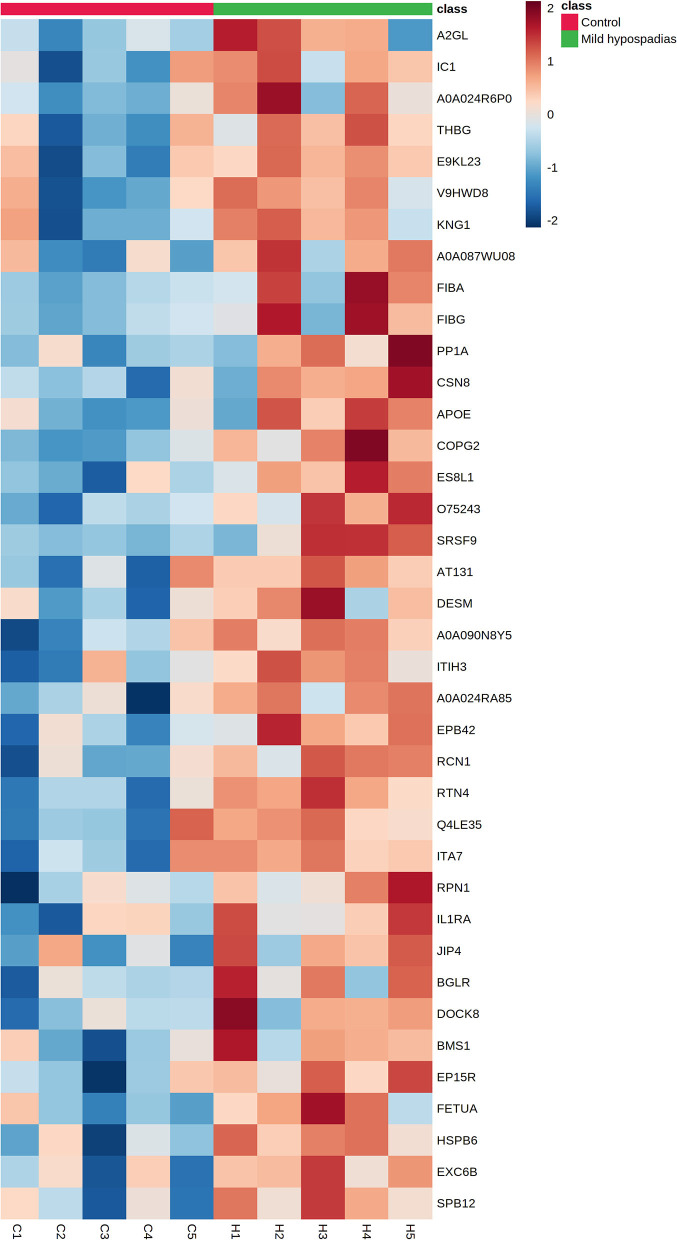
Proteins found upregulated in the proteome from mild hypospadias patients. Thirty-eight proteins were found increased between control and mild hypospadias samples. Heatmaps depict protein abundance changes in terms of Zq across samples (C1-C5 for control and H1-H5 for mild hypospadias samples). The magnitude of abundance change [increased (red) or decreased (blue)] is shown according to the color scale on the right.

A multiplexed, quantitative proteomics approach based on 10-plex TMT labeling and LC-MS/MS analysis was employed to identify and quantify proteins of foreskin tissue from mild hypospadias and control samples (*n* = 5/group). A total of 2,522 proteins were identified and quantified with more than one unique peptide. Multivariate analyses revealed differences between the proteome of mild hypospadias samples when compared to control samples. Principal component analysis showed that control samples displayed a wide distribution, whereas samples from mild hypospadias patients were similar amongst each other. Components were explained by principal component (PC) 1 with a 29.2% variance and PC 2 with a 18.1% variance. Figure 2A depicts PC1 and PC2 components with 95% confidence ellipses for control and mild hypospadias samples. Furthermore, PLS-DA showed a marked separation between control and mild hypospadias groups. Partial least squares (PLS) 1 contributed 17.5%, whereas PLS2 carried 21.9% of the total variance. Figure 2B shows the separation between control and mild hypospadias groups with 95% confidence ellipses. One hundred thirty-three proteins were identified with significant changes in protein abundance in mild hypospadias samples in comparison to control samples at 1% FDR (available at PeptideAtlas repository). Specifically, 95 proteins were found decreased (Figure 3), and 38 proteins were increased (Figure 4) in mild hypospadias when compared to control samples. Supplementary Table 1 details accession numbers, gene, and protein names for these proteins.

### Enriched Biological Processes in Mild Hypospadias

We performed an unbiased functional enrichment analysis for proteins showing significantly decreased or increased abundances using STRING network analysis. Interacting proteins were found in both sets of proteins with a PPI enrichment *p*-value < 1.0e-16. For proteins with decreased levels, 257 Gene Ontology (GO) terms were found significantly enriched (Supplementary Table 2). For proteins with increased levels, 79 GO terms were found significantly enriched (Supplementary Table 3). Within the identified biological processes that were enriched, we observed pathways involving cellular energy processes and apoptotic signaling (Table 2).

**Table 2:** Enriched functional categories involving cellular energy processes and apoptotic signaling pathways.

**GO term**	**Description**	**False discovery rate (FDR)**	**Proteins in gene set**
**Gene Ontology (GO)**			
GO:0006091	Generation of precursor metabolites and energy	0.00000326	ADH1A, ADH1B, ATP5J, CYCS, FH, GPD2, HK1, NDUFAB1, NNT, OGDH, PGK1, SUCLA2
GO:0045333	Cellular respiration	0.00012	CYCS, FH, GPD2, NDUFAB1, NNT, OGDH, SUCLA2
GO:0046034	ATP metabolic process	0.00029	ATP1B1, ATP5J, CYCS, HK1, NDUFAB1, OGDH, PGK1
GO:0006099	Tricarboxylic acid cycle	0.00043	FH, NNT, OGDH, SUCLA2
GO:0006096	Glycolytic process	0.0067	HK1, OGDH, PGK1
GO:0006094	Gluconeogenesis	0.0089	GOT2, GPD2, PGK1
GO:0097193	Intrinsic apoptotic signaling pathway	0.0206	AIFM1, CUL3, CUL4A, CYCS
GO:0022904	Respiratory electron transport chain	0.0149	CYCS, GPD2, NDUFA6, NDUFAB1
GO:0006754	ATP biosynthetic process	0.0081	ATP5J, HK1, OGDH, PGK1
GO:2000352	Negative regulation of endothelial cell apoptotic process	0.0078	FGA, FGG
GO:1902042	Negative regulation of extrinsic apoptotic signaling pathway *via* death domain receptors	0.0108	FGA, FGG

### Cytochrome C (CYCS) Validation

**Figure 5:**
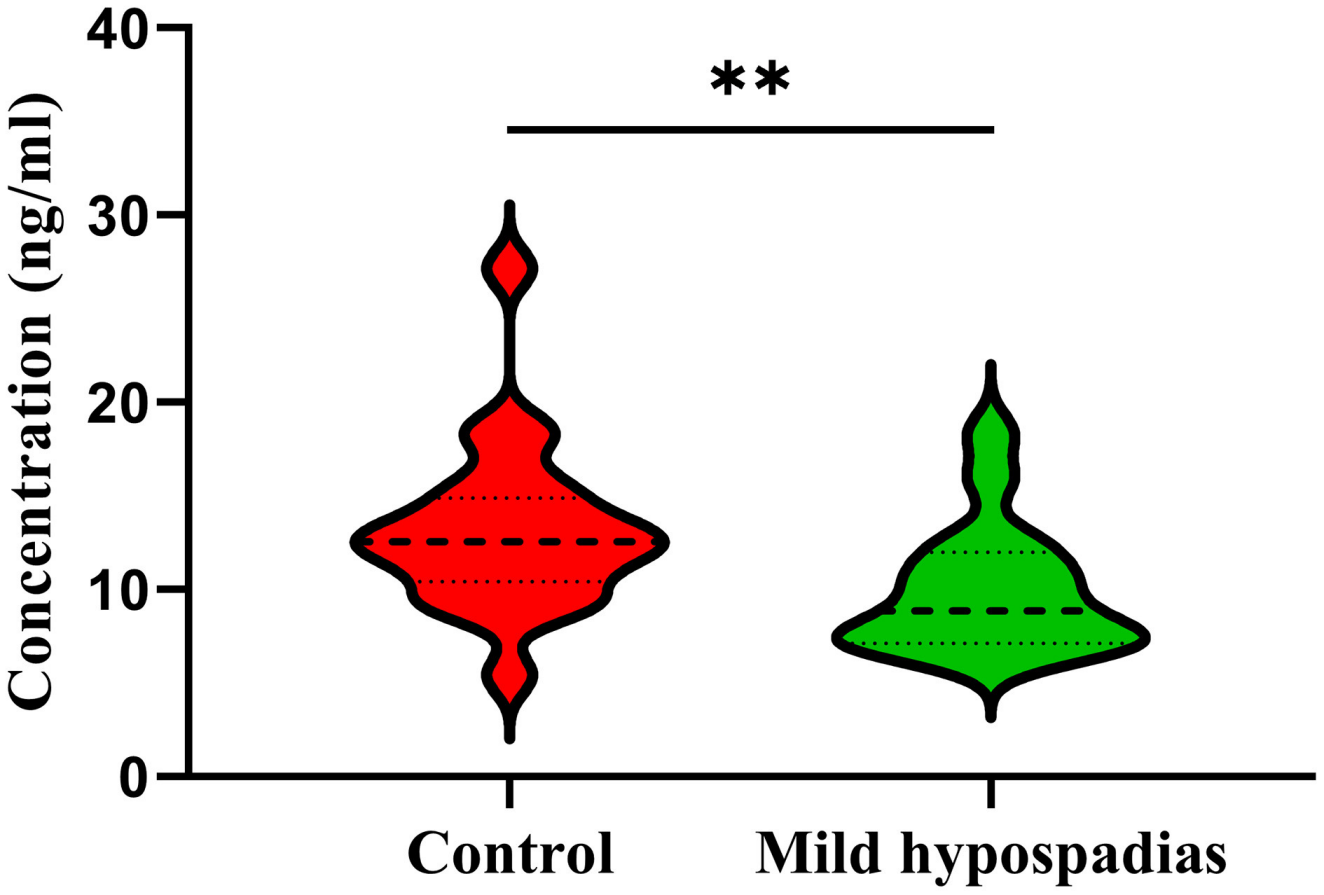
Validation of cytochrome C by ELISA. Cytochrome C was found in significantly lower concentrations in mild hypospadias samples (median = 8.86 ng/ml) in comparison to controls (median = 12.56 ng/ml) samples in agreement with proteomics results. Dashed line represents the median value. Dotted lines represent quartiles, top: 75% percentile and bottom: 25% percentile. ** = *p* < 0.05. Controls, *n* = 10; Mild hypospadias, *n* = 8.

CYCS is an essential protein in the mitochondrial electron transport chain that also plays key roles in apoptosis through the caspase-dependent apoptosis pathway. Given that biological processes related to cellular energy and apoptotic processes were found significantly enriched in this study, CYCS was selected for validation with ELISA. Significant differences between control and mild hypospadias samples were found (*p* = 0.0067). Specifically, CYCS was found in lower concentrations (ng/ml) in mild hypospadias (median = 8.86) when compared to control samples (median = 12.56; Figure 5).

## Discussion

To the best of our knowledge, this is the first study that has evaluated the proteome in mild hypospadias, which is the most common type of this congenital urogenital condition accounting for nearly 70% of cases. We have analyzed human foreskin samples by using a multiplexed, quantitative proteomics approach based on SIL and LC-MS/MS that has allowed the identification of 2,522 proteins which were identified with two or more unique peptides. Significant differences in protein abundances were identified for 133 proteins. Specifically, 95 proteins were found with significantly decreased protein abundance and 38 with significantly increased protein abundance in mild hypospadias when compared to control samples. It is of interest that unbiased functional enrichment analysis revealed mitochondrial energy production and apoptotic signaling pathways as significantly enriched processes.

Mitochondrial dysfunction has been linked to congenital conditions such as spina bifida, cardiomyopathy, and inborn errors of metabolism (58–60). Our enrichment data, from known and predicted protein-protein interactions, revealed interactions between *ADH1A, ADH1B, ATP5J, CYCS, DERA, FH, GPD2, HK1, NDUFAB1, NDUFA6, NNT, OGDH, PGK1*, and *SUCLA2* for generation of precursor metabolites and energy; *CYCS, FH, GPD2, NDUFAB1, NDUFA6, NNT, OGDH*, and *SUCLA2* for cellular respiration; *CYCS, GPD2, NDUFA6*, and *NDUFAB1* for respiratory electron transport chain; *ATP1B1, ATP5J, CYCS, HK1, NDUFAB1, NDUFA6, OGDH*, and *PGK1* for ATP metabolic process; *FH, NNT, OGDH*, and *SUCLA2* for tricarboxylic acid cycle; *ATP5J, HK1, OGDH*, and *PGK1* for ATP biosynthetic process; *HK1, OGDH*, and *PGK1* for glycolytic process; and *GOT2, GPD2*, and *PGK1* for gluconeogenesis, among other which indirectly contribute to cellular energy processes such as glucose metabolic process, fatty acid metabolic process, and fumarate metabolic process.

Embryonic development demands high energy inputs to carry out essential processes such as cellular differentiation, cell migration, proliferation, and patterning processes. Early during the embryonic period, the major source of energy is glycolysis. As the embryo develops, cells produce energy from the three major processes of energy production in eukaryotic cells: glycolysis, Krebs cycle, and the electron transport chain (61). Thus, it is not surprising that the dysregulation of proteins involved in the production of cellular energy demands has been linked to congenital conditions, as shown here. For instance, *PGK, NNT, SUCLA2*, and *FH* have been linked to inherited disorders including, but not limited to, infantile encephalopathy, X-linked inherited disorders, myopathies, and neural tube defects (62–66). Programmed cell death is also an energy-dependent process, and, as reported here, the intrinsic apoptotic signaling pathway (67, 68) was disrupted in mild hypospadias samples. Taken together, our results may present a new molecular research strategy to study mild hypospadias, although further research on the involvement of mitochondrial dysfunction in this congenital condition may provide promising venues for future scientific inquiry.

Furthermore, apoptosis plays a key role during the development of the male genitalia (51, 69, 70). Previous studies have proposed that the suppression of apoptotic-related genes can give rise to hypospadias (71, 72). For instance, reduced apoptosis is observed in finasteride-induced hypospadiac rats through the MAPK/ERK signaling pathway (73). In addition, as confirmed by the presence of apoptotic cells, the luminization of the urethra in human embryo specimens is accomplished by apoptosis (51).

Of note, in this study, *CYCS* was found significantly downregulated in mild hypospadias samples. *CYCS* is an inner mitochondrial protein with key biological roles in mitochondrial electron transport and apoptosis. Cytochrome C assumes an apoptotic role when released into the cytosol. Suppression or activation of anti- and pro-apoptotic members of the *Bcl-2* family leads to altered mitochondrial membrane permeability resulting in the release of *CYCS* into the cytosol. Once in the cytosol, *CYCS* binds to *Apaf-1*, which triggers the activation of caspase-9, which in turn accelerates apoptosis by the activation of other caspases (74). Given the major role of *CYCS* as a modulator of apoptosis, we analyzed additional samples by ELISA, which revealed a significant downregulation of *CYCS* in mild hypospadias as compared to control samples. It remains as a challenge for future experiments to determine the time course of such downregulation across development.

*AIFM1*, which has also been found downregulated in the hypospadias group, is a pro-apoptotic factor in the caspase-independent pathway, which, in response to apoptotic stimuli, is released from the mitochondrial intermembrane space into the cytosol and to the nucleus (74). Mutations of *AIF* can lead to severe mitochondrial dysfunction and have been associated with several disorders and neuropathogenesis (75–77). *CUL3* and *CUL4A*, which were also downregulated, are part of the Cullin gene family, the largest ubiquitin ligase family in mammals, which are part of the core component of multiple cullin-RING-based E3 ubiquitin-protein ligase complexes that mediate the ubiquitination of target proteins and subsequent proteasomal degradation. These proteins participate in the extrinsic apoptotic pathway activated by the tumor necrosis factor (*TNF*) receptor family through the ubiquitination of caspase-8 (78–80). *CUL4A*-mediated protein degradation has been reported as essential for the maturation of primary spermatocytes in mouse testis (81). Taken together, the observed downregulation of *AIFM1, CUL3, CUL4A*, and *CYCS* in this study warrants further research.

There are a number of limitations to this study. As the first study of the proteome in hypospadias, it is necessary to replicate the results presented here with a larger patient cohort. Owing to the difficulties inherent to obtaining patient tissue samples, the high cost of proteomics analysis, and the limited accession to instruments, many clinical proteomics studies are based on relatively small patient cohorts [see, for example, (82–84)]. It is important to validate clinical proteomics results with larger patient cohorts and by using orthogonal techniques like ELISA and Western blot. The validation of Cytochrome C presented here is a first step in this direction. It must be noted, however, that in this study, the limited number of samples is compensated by the application of WSPP as a rigorous statistical model. Second, although the current state of knowledge on the proximal growth of the preputial lamina over the glans penis is most-likely an androgen-dependent effect that is completed during intrauterine development (85), this study cannot address whether the so-called “mini-puberty” in males (86, 87) contributes further to cytoarchitectural changes of foreskin samples so that some of the changes in protein levels observed here could be due, at least in part, to postnatal androgen-dependent effects in foreskin tissue up to 12 months of age. Important to note is that available evidence suggests that human penile growth is androgen-independent from ~12 months of age up to puberty (87). Given the age range of our patient cohort, we cannot address this question. Nevertheless, in humans, the effects of postnatal cytoarchitectural changes on foreskin protein expression will most likely remain as a *vexata quaestio* as it is unethical to time urethroplasty procedures to address this experimental conundrum. Related to this, the third limitation of this study is the significant age difference between experimental groups. Although it has been noted that histological and morphological changes occur in foreskin tissue during postnatal development (88), such as changes in vasculature, connective tissue, and nervous tissue, for example, we are of the opinion that it is unlikely that such structural changes during development heavily rely on the distinct cellular energy and apoptotic pathways reported here. The fourth limitation of this study is related to the histological origin of foreskin samples, which were mostly comprised of lateral and dorsal foreskin primarily due to the ventral deficiency that characterizes hypospadias. This study cannot rule out the possibility of under-or over-estimating the levels of protein expression because it is not possible to sample the circumferential prepuce in hypospadias. Of interest, the formation of the circumferential prepuce during intrauterine development is an androgen-independent event (89). In fact, future studies on the dependency of androgen signaling for the changes in protein expression levels observed here may provide valuable insights on the timing of embryological events that are affected in hypospadias.

The results reported in this study rely on a multi-step data analysis approach within a carefully designed statistical framework that allows for the identification of proteins, their relative abundances, and the quantitative analysis of whether such abundances significantly differ among experimental groups. It is important to underscore that pathways and protein-protein interactions reported here rely on existing protein databases. These are “predicted” pathways and interactions based on published data from a myriad of biological systems studied with a staggering diversity of experimental approaches. The ones discussed here are based on our own interpretation or previously linked to hypospadias or other congenital conditions.

In conclusion, this first line of proteomics assessment shows a differential proteome profile for mild hypospadias. Proteins related to cellular energy production and apoptotic pathways stand out in this proteomics signature. These pathways may facilitate the search for novel candidates in the etiology of mild hypospadias and may also constitute potential biomarkers for early detection of this congenital urogenital condition.

## Data Availability Statement

The datasets generated in this study can be found in online repositories. The names of the repository/repositories and accession number(s) can be found below: http://www.peptideatlas.org/PASS/PASS01602.

## Ethics Statement

The studies involving human participants were reviewed and approved by Human Research Subjects Protection Office (HRSPO) at the University of Puerto Rico, Medical Sciences Campus. Written informed consent to participate in this study was provided by the participants' legal guardian/next of kin.

## Author Contributions

The study was designed by CP-R, MP-B, and JJ. MP-B performed all surgical procedures and collected all tissue samples. CP-R assisted in sample collection and conducted sample preparation for proteomics experiments under the guidance and expert advice of HS and conducted validation experiments. IJ, EC, RM, and JV developed statistical algorithms and submitted collected samples to proteomic analyses. EM-V assisted in sample preparation and performed statistical analysis for validation experiments. Final analyses, the depiction of data, and writing of the first draft were by CP-R and JJ. All authors participated in the drafting and approval of the final version of the manuscript.

## Conflict of Interest

The authors declare that the research was conducted in the absence of any commercial or financial relationships that could be construed as a potential conflict of interest.
