# Supplementary material for: A Proteomics Signature of Mild Hypospadias: A Pilot Study
Source: Front Pediatr. 2020 Dec 23;8:586287. doi: 10.3389/fped.2020.586287 (PMC7786202; doi:10.3389/fped.2020.586287)
Supplement: Supplementary file 1 [file Table_1.pdf]

Supplementary Material

1 Supplementary Tables

Supplementary Table 1. List of the 133 proteins that showed significant abundance changes between control and mild hypospadias samples. The table provides the accession number, gene, and protein names.

Accession #	Gene name	Protein
Q13200	PSMD2	26S proteasome non-ATPase regulatory subunit 2
P62333	PRS10	26S proteasome regulatory subunit 10B
P62195	PRS8	26S proteasome regulatory subunit 8
Q02218	ODO1	2-oxoglutarate dehydrogenase, mitochondrial
O14561	ACPM	Acyl carrier protein, mitochondrial
A0A024R497	A0A024R497	Acyl-CoA synthetase long-chain family member 3, isoform CRA
P30520	PURA2	Adenylosuccinate synthetase isozyme 2
P07327	ADH1A	Alcohol dehydrogenase 1A
P00325	ADH1B	Alcohol dehydrogenase 1B
P02765	FETUA	Alpha-2-HS-glycoprotein
Q12904	AIMP1	Aminoacyl tRNA synthase complex-interacting multifunctional protein 1
O95782	AP2A1	AP-2 complex subunit alpha-1
O94973	AP2A2	AP-2 complex subunit alpha-2
P02649	APOE	Apolipoprotein E
O95831	AIFM1	Apoptosis-inducing factor 1, mitochondrial
P00505	AATM	Aspartate aminotransferase, mitochondrial
Q6IB54	Q6IB54	ATP synthase-coupling factor 6, mitochondrial
P28288	ABCD3	ATP-binding cassette sub-family D member 3
P53396	ACLY	ATP-citrate synthase
A0A024R3Z6	A0A024R3Z6	Basic leucine zipper and W2 domains 1, isoform CRA
P08236	BGLR	Beta-glucuronidase
P07814	SYEP	Bifunctional glutamate/proline--tRNA ligase
P63098	CANB1	Calcineurin subunit B type 1
P21926	CD9	CD9 antigen
A0MNN4	A0MNN4	CDW3/SMU1
A0A024RA85	A0A024RA85	Cell division cycle 2-like 5 (Cholinesterase-related cell division controller), isoform CRA
B3KTR3	B3KTR3	Chloride intracellular channel protein
O60271	JIP4	C-Jun-amino-terminal kinase-interacting protein 4
Q9UBF2	COPG2	Coatomer subunit gamma-2 OS=Homo sapiens
O60826	CCD22	Coiled-coil domain-containing protein 22

Q7L5N1	CSN6	COP9 signalosome complex subunit 6
Q99627	CSN8	COP9 signalosome complex subunit 8
P78310	CXAR	Coxsackievirus and adenovirus receptor
Q13618	CUL3	Cullin-3
Q13619	CUL4A	Cullin-4A
P99999	CYC	Cytochrome c
Q14008	CKAP5	Cytoskeleton-associated protein 5
Q8NF50	DOCK8	Dedicator of cytokinesis protein 8
Q9Y315	DEOC	Deoxyribose-phosphate aldolase
P17661	DESM	Desmin
P32926	DSG3	Desmoglein-3
P11388	TOP2A	DNA topoisomerase 2-alpha
O95602	RPA1	DNA-directed RNA polymerase I subunit RPA1
E5KT65	E5KT65	DNA-directed RNA polymerase subunit RPABC1
P04843	RPN1	Dolichyl-diphosphooligosaccharide--protein glycosyltransferase subunit 1
P50570	DYN2	Dynamin-2
Q96J02	ITCH	E3 ubiquitin-protein ligase Itchy homolog
P42126	ECI1	Enoyl-CoA delta isomerase 1, mitochondrial
Q8TE68	ES8L1	Epidermal growth factor receptor kinase substrate 8-like protein 1
Q9UBC2	EP15R	Epidermal growth factor receptor substrate 15-like 1
V9HW51	V9HW51	Epididymis secretory protein Li 114
V9HWD8	V9HWD8	Epididymis secretory sperm binding protein Li 163pA
E9KL23	E9KL23	Epididymis secretory sperm binding protein Li 44a
V9HWE8	V9HWE8	Epididymis secretory sperm binding protein Li 47e
P16452	EPB42	Erythrocyte membrane protein band 4.2
O00303	EIF3F	Eukaryotic translation initiation factor 3 subunit F
Q9UBQ5	EIF3K	Eukaryotic translation initiation factor 3 subunit K
Q6IS14	IF5AL	Eukaryotic translation initiation factor 5A-1-like
Q9Y2D4	EXC6B	Exocyst complex component 6B
O14980	XPO1	Exportin-1
Q92945	FUBP2	Far upstream element-binding protein 2
P02671	FIBA	Fibrinogen alpha chain
P02679	FIBG	Fibrinogen gamma chain
P07954	FUMH	Fumarate hydratase, mitochondrial
O94925	GLSK	Glutaminase kidney isoform, mitochondrial
Q6FII1	Q6FII1	Glutathione S-transferase kappa
P43304	GPDM	Glycerol-3-phosphate dehydrogenase, mitochondrial
A0A087WU08	A0A087WU08	Haptoglobin
Q9H583	HEAT1	HEAT repeat-containing protein 1
O14558	HSPB6	Heat shock protein beta-6 OS=Homo sapiens

A0A024RDF6	A0A024RDF6	Heterogeneous nuclear ribonucleoprotein D-like, isoform CRA
P19367	HXK1	Hexokinase-1
A0A0S2Z410	A0A0S2Z410	Hydroxysteroid dehydrogenase 10 isoform 1 (Fragment)
Q14974	IMB1	Importin subunit beta-1
O95373	IPO7	Importin-7
Q13683	ITA7	Integrin alpha-7
Q06033	ITIH3	Inter-alpha-trypsin inhibitor heavy chain H3
P18510	IL1RA	Interleukin-1 receptor antagonist protein
Q4LE35	Q4LE35	ITGA7 variant protein (Fragment)
P01042	KNG1	Kininogen-1
Q9UIC8	LCMT1	Leucine carboxyl methyltransferase 1
P02750	A2GL	Leucine-rich alpha-2-glycoprotein
A0A090N8Y5	A0A090N8Y5	LSM5 homolog, U6 small nuclear RNA associated (S. cerevisiae)
Q9HD20	AT131	Manganese-transporting ATPase 13A1
O14880	MGST3	Microsomal glutathione S-transferase 3
O96008	TOM40	Mitochondrial import receptor subunit TOM40 homolog
O95202	LETM1	Mitochondrial proton/calcium exchanger protein
L7RXH5	L7RXH5	Mitogen-activated protein kinase
Q8NFW8	NEUA	N-acylneuraminate cytidylyltransferase
P56556	NDUA6	NADH dehydrogenase [ubiquinone] 1 alpha subcomplex subunit 6
A0A024R0C3	A0A024R0C3	Nicotinamide nucleotide transhydrogenase, isoform CRA
A0A024R6W2	A0A024R6W2	Nudix (Nucleoside diphosphate linked moiety X)-type motif 21, isoform CRA
Q8TBX8	PI42C	Phosphatidylinositol 5-phosphate 4-kinase type-2 gamma
P00558	PGK1	Phosphoglycerate kinase 1
P05155	IC1	Plasma protease C1 inhibitor
O43143	DHX15	Pre-mRNA-splicing factor ATP-dependent RNA helicase DHX15
A0A0S2Z489	A0A0S2Z489	Proteasome (Prosome, macropain) 26S subunit, non-ATPase, 12
A0A109NGN6	A0A109NGN6	Proteasome subunit alpha type
P60903	S10AA	Protein S100-A10
P55786	PSA	Puromycin-sensitive aminopeptidase
O75243	O75243	R30783_1
A0A024R1U4	A0A024R1U4	RAB5C, member RAS oncogene family, isoform CRA
A0A140VK94	A0A140VK94	RAN binding protein 1, isoform CRA
Q86VI3	IQGA3	Ras GTPase-activating-like protein IQGAP3
P60763	RAC3	Ras-related C3 botulinum toxin substrate 3
D6R9Z1	D6R9Z1	Receptor of-activated protein C kinase 1 (Fragment)
Q15293	RCN1	Reticulocalbin-1
Q9NQC3	RTN4	Reticulon-4 OS=Homo sapiens

O75116	ROCK2	Rho-associated protein kinase 2
Q14692	BMS1	Ribosome biogenesis protein BMS1 homolog
P49756	RBM25	RNA-binding protein 25
Q9Y230	RUVB2	RuvB-like 2
Q13242	SRSF9	Serine/arginine-rich splicing factor 9
Q13177	PAK2	Serine/threonine-protein kinase PAK 2
P62136	PP1A	Serine/threonine-protein phosphatase PP1-alpha catalytic subunit
Q96P63	SPB12	Serpin B12
A0A024R6P0	A0A024R6P0	Serpin peptidase inhibitor, clade A (Alpha-1 antiproteinase, antitrypsin), member 3, isoform CRA
P61009	SPCS3	Signal peptidase complex subunit 3
Q04837	SSBP	Single-stranded DNA-binding protein, mitochondrial
P05026	AT1B1	Sodium/potassium-transporting ATPase subunit beta-1
Q15459	SF3A1	Splicing factor 3A subunit 1
Q13435	SF3B2	Splicing factor 3B subunit 2
P26368	U2AF2	Splicing factor U2AF 65 kDa subunit
P38646	GRP75	Stress-70 protein, mitochondrial
Q14683	SMC1A	Structural maintenance of chromosomes protein 1A
E5KS60	E5KS60	Succinate--CoA ligase [ADP-forming] subunit beta, mitochondrial
O60264	SMCA5	SWI/SNF-related matrix-associated actin-dependent regulator of chromatin subfamily A member 5
P78371	TCPB	T-complex protein 1 subunit beta
P05543	THBG	Thyroxine-binding globulin
Q14232	EI2BA	Translation initiation factor eIF-2B subunit alpha
Q93009	UBP7	Ubiquitin carboxyl-terminal hydrolase 7
A0A024R1P7	A0A024R1P7	Unc-84 homolog B (C. elegans), isoform CRA
Q548N1	Q548N1	Vacuolar protein sorting-associated protein 28 homolog

Supplementary Table 2. Functional enrichment analysis for decreased proteins. Unbiased functional enrichment analysis was performed for proteins with significant increased abundance using STRING network analysis. STRING database (http://string-db.org), version 11.0, © STRING CONSORTIUM 2020, was used for functional enrichment analysis. Please, download Table 2 file.

Supplementary Table 3. Functional enrichment analysis for increased proteins. Unbiased functional enrichment analysis was performed for proteins with significant decreased abundance using STRING network analysis. STRING database (http://string-db.org), version 11.0, © STRING CONSORTIUM 2020, was used for functional enrichment analysis. Please, download Table 3 file.
